# Modification of Higher Alkanes by Nanoparticles to Control Light Propagation in Tapered Fibers

**DOI:** 10.3390/mi11111006

**Published:** 2020-11-14

**Authors:** Karol A. Stasiewicz, Iwona Jakubowska, Joanna Korec, Katarzyna Matras-Postołek

**Affiliations:** 1 Institute of Applied Physics, Faculty of Advanced Technologies and Chemistry, Military University of Technology, 2 Kaliskiego St., 00-908 Warsaw, Poland; iwona.jakubowska@wat.edu.pl (I.J.); joanna.korec@wat.edu.pl (J.K.); 2 Department of Biotechnology and Physical Chemistry, Faculty Chemical Engineering and Technology, Cracow University of Technology, 24 Warszawska St., 31-155 Cracow, Poland; matras@chemia.pk.edu.pl

**Keywords:** nanoparticles, optical fiber taper, fiber technology, sensors, higher alkanes, advancements in fiber forming technologies, functional micro- and nanofibers for miniaturisation technologies

## Abstract

This study presents the doping of higher alkanes, namely, pentadecane (C15) and hexadecane (C16), with ZnS:Mn nanoparticles to create new types of in-line optical fiber sensors with unique optical properties. In this research, the phenomenon of light beam leakage out of the taper and its interaction with the surrounding materials is described. The fabricated new materials are used as cladding in a tapered optical fiber to make it possible to control the optical light beam. The manufactured sensor shows high sensitivity and fast response to the change in the applied materials. Results are presented for a wide optical range of 1200–1700 nm with the use of a supercontinuum source and an optical spectrum analyzer, as well as for a single wavelength of 800 nm, corresponding to the highest transmitted power. The results present a change in the optical property dependence on the temperature in the cooling and heating process. For all materials, the measurements in a climatic chamber are provided between 0 and 40 °C, corresponding to the phase change of the alkanes from solid to liquid. The addition of nanoparticles to the volume of alkanes is equal to 1 wt%. To avoid a conglomeration of nanoparticles, the anti-agglomeration material, Brij 78 P, is used.

## 1. Introduction

In recent years, a rapid development in optical fiber technology has been observed [1]. Light propagation in a fiber is associated with the difference between the fiber structure, especially the properties of the core and cladding materials, corresponding in most cases with their refractive indices *n_core_* and *n_cladding_*, and the core diameter. For all kinds of fibers, a fundamental requirement is to protect the light inside the structure. The electromagnetic field of light waves propagating inside the fiber can be derived from solving Maxwell’s equations with boundary conditions given by the material structure [1,2,3]. Two methods of light propagation are currently possible. The first method is based on Snell’s law, where the total internal reflection (TIR [3]) phenomenon is used. This method of propagation is mostly used for standard fibers with a solid core and cladding, and with an mTIR modified version for photonic crystal fibers with the structure of air holes representing the cladding and solid core. The second method of light propagation involves the phenomenon of photonic bandgap propagation [4], which is mostly used in a photonic crystal fibers with air hole cores.

In standard fibers, the difference between the value of the refractive index of the core and cladding determines the number of modes propagating in a fiber, known as the normalized frequency *V*, which is equal to 2405. This parameter is described by [5]:

(1) $$V=a\frac{2\pi }{\lambda }\sqrt{n_{core}{}^{2}-n_{cladding}{}^{2}}$$

Additionally, this parameter is dependent on the wavelength and the core diameter. To change the propagation properties in both cases, it is necessary to change the refractive index or the core diameter.

A second method for changing the light propagation parameter is to modify the size of the fiber core. It should be noted that the propagation of a light beam in fibers, especially in a standard one, due to Snell’s law, is also connected with a reflection on the border of two materials from which the core and cladding are formed. In such a propagation, part of the power passes from the core to the cladding for a certain distance. This distance is known as the penetration depth of light from the core to the cladding and can be described by [6,7,8]:

(2) $$d_{p}=\frac{\lambda }{2\pi \sqrt{n_{core}{}^{2}\sin^{2}\theta -n_{cladding}{}^{2}}}$$

where *λ* is the determined wavelength of the light, *θ* is the angle of the incident plane wave on the core/cladding surface and *n_core_* and *n_cladding_* are the core and cladding refractive indices, respectively.

This parameter depends on wavelength propagation in fibers, as well as on the angle of an incident plane wave on the core and cladding border [8,9,10]. It is noteworthy that this parameter enables direct access to the light and increases the sensitivity to external changes in the environment. In the presented case, the applied alkanes with nanoparticles interact in a leaking light as double-clad materials that change their phase state.

Theoretical and experimental investigation of changing core and cladding diameters, optical beam parameters, as well as a refractive index profile along with the fiber show that with the reduction of the diameter/dimensions of the core and the clading, the diameter of the propagating beam increases, filling the entire taper structure and extending beyond it [11].

For both kinds of fibers, a technique known as tapering can be applied for changing the core and cladding diameters [6,7,8,9,10,11]. Tapering technology uses a relatively easy technique to fill the air holes and provides the possibility of the continuous monitoring of changes of the light propagation through the fiber during element manufacturing, i.e., a diameter of taper region control.

The above description of light propagation in different fibers shows its wide range of applications. The telecommunication application of fibers is widely described in the literature [12]. Nowadays, significant interest is directed to optical fiber sensors [12,13] due to their small dimensions, low weight, rapid response, and the possibility of operating in different environments. Their construction is divided into several groups. One of the groups is based on an interferometry measurement that enables very precise measurements [13] but requires an advanced system of acquisition and causes an increase in dimensions. Some sensors use fibers as elements that deliver light to the measurement places and their light beam is output from the fiber and interacts with a measurement factor [14]. The final part of sensors uses the change of light amplitude as an interaction of the external parameters of the fiber [14]. The accuracy and sensitivity of such sensors are strictly connected with the influence of the detected factors on light propagation. For these reasons, in many detectors, additional materials are introduced as media that multiply the signal level, which influences the light beam. In all the above mentioned cases, the main advantage is that the light measures the change of the surrounding environment without changing the material and geometrical properties of the investigated material.

In addition, in recent years, nanoparticles have become a significant topic of interest. Unique results for light interaction with matter can be observed in semiconducting nanoparticles. They possess interesting optical, electronic, chemical, and electrochemical properties that are useful for a wide variety of applications, including optoelectronics [15,16], catalysis [17,18] and sensing [19]. Among II-VI semiconducting nanoparticles, manganese-doped zinc sulfide (ZnS:Mn) nanocrystals have been substantially studied due to their unique properties and their diverse applications, including photovoltaics [20,21] and transparent nanocomposites [22]. Zinc sulfide is a large band-gap energy (3.6 eV) semiconductor that is well known for its photoluminescence, electroluminescence, and thermoluminescence. ZnS:Mn and its orange emission have been known since the early days of luminescence research because of its high quantum efficiency at room temperature. ZnS:Mn is non-toxic, chemically more stable, and technologically better than many other semiconducting materials based on toxic elements, such as CdSe and CdS [23,24].

In this study, threshold temperature sensors based on the use of additional materials, namely, alkanes with a nanoparticle admixture that influence light propagation in a biconical fiber taper, are presented.

## 2. Materials and Methods

### 2.1. Materials

**Figure 1:**
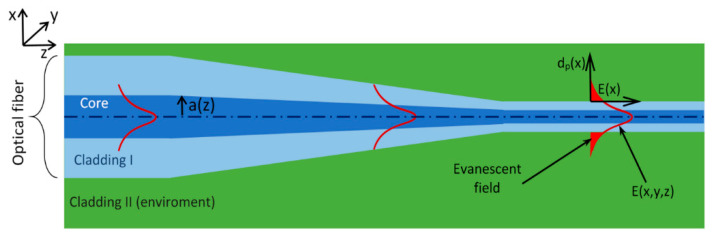
Propagation of light in an optical fiber taper [25,26].

In the research, a standard optical fiber (SMF28e) was used and built as two concentrically arranged materials to form the structure of core and cladding (Figure 1).

Table 1 presents the main parameters of the used fiber in the future technological process. As can be seen, the refractive index of the core is slightly higher than the refractive index of the cladding. Some part of the energy leaks to the cladding as an evanescence field (Figure 1) within a distance of up to several dozen nm [10], known as the penetration depth. In such a structure, there are no possibilities to directly interact with the light propagating inside the core due to the much larger dimensions of the thickness of the fiber cladding.

One of the methods used to obtain a decreasing diameter of the core and the cladding is a process in which part of the fiber is heated to the melting point and is then stretched out. This technique is called the tapering technique. Due to the wide description of tapers in literature, hence, it will not be presented in this paper [7,10].

The obtained structure can be described as a structure in which the light propagates in a dielectric structure (taper waist) corresponding to the fiber core and the air surrounding becomes the cladding. The difference in refractive indices between the core and the cladding in a standard fiber is ~1% (see Table 1). For a structure with surrounding air (refractive index of ~1003), the value is ~45% (assuming the average value of the collapse coverage made the structures 1.45). In such arrangements, there exists an opportunity to replace air with various materials. Such works have been widely used to build sensors, filters, amplifiers [27,28,29,30,31,32,33,34,35].

As base materials that act as double cladding, higher alkanes, such as n-pentadecane (C15) and n-hexadecane (C16), have been applied. Table 2 shows the main parameters of these alkanes [36,37].

The presented chemical properties of the used materials were checked in our laboratories at a temperature of 20.4 °C using a Hanna Instruments HI 968000 (Woonsocket, Rhode Island, USA) electronic refractometer in the range of 1.3330–1.5040 nD20, with a control material standard water used for the measurement of the refractive index equal to 1.3329.

These materials are very well known and have been described widely [38,39,40]. As seen from Table 2, the refractive indices are close to those of the standard cladding of the fiber.

It should be mentioned that higher alkanes change their transparency for light together with phase change in a melting point [41,42]. Analyzing the state of RI in a solid stated, the extinction ratio is high, which also causes that the real part of RI of alkanes is higher than RI of fibers which influences the light attenuation. 

**Figure 2:**
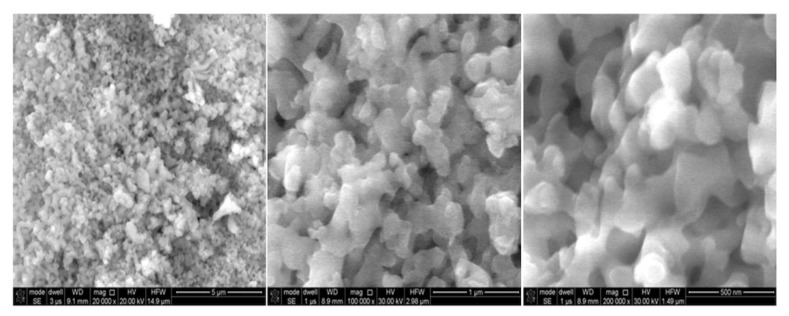
Picture of applied nanoparticles of ZnS:Mn + DBSA + CYS.

**Figure 3:**
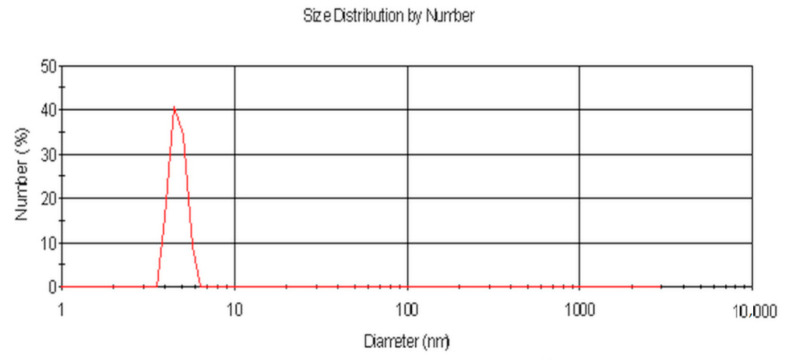
Particle size distribution of ZnS:Mn nanoparticles stabilised by CYS and DBSA in tetrahydrofuran.

The innovation in this study is an admixture of the mentioned alkanes with nanoparticles of ZnS:Mn + DBSA + CYS, which means that nanoparticles consist of the material ZnS: Mn (zinc sulfide: manganese) and have a combination of dodecylbenzenesulfonic acid (DBSA) and 2-mercaptoethylamine hydrochloride (cysteaminium chloride, cys determination) on the surface. Images of the used nanoparticles are presented in Figure 2. Figure 3 shows the particle size distribution of the ZnS:Mn nanoparticles.

**Figure 4:**
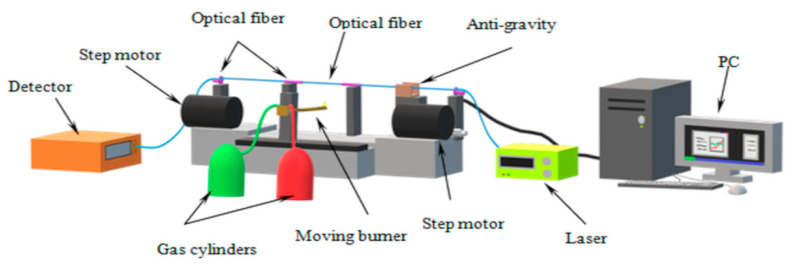
Schematic of FOTET system for biconical optical fiber manufacturing [34].

The size of the ZnS:Mn nanoparticles dispersed in THF (Tetrahydrofuran) was determined by DLS (dynamic light scattering). Figure 4 shows the resulting distribution, with a hydrodynamic radius of ~6 nm. According to the Scherrer equation, the calculated crystallite size of the particles is three times smaller than the diameter, as determined by dynamic light scattering. This can be explained by the intensity weighted indication of the particle diameter and the organic shell around the particle, leading to higher values for the particle diameters. Additionally, it has to be considered that crystal imperfections contribute to additional line broadening in X-ray diffraction. 

The materials for the synthesis of the ZnS:Mn nanoparticles were used without additional purifications. Manganese (II) acetate tetrahydrate (~99%), zinc (II) acetate dehydrate (~98%), and tetratrahydrofuran (~99%) were purchased from Sigma Aldrich. Cysteamine chloride (~97%) and 4-dodecylbenzenesulphonic acid (~90%) were purchased from Merck and Fluka, respectively. Sodium sulfide hydrate (~60%) was pursed from Riedel-de Haan. The synthesis procedure for the ZnS:Mn nanoparticles is patented [27]. The ZnS:Mn nanoparticles were stabilised by cysteaminium chloride and 4-dodecylbenzylsulphonic acid were used in our previous work as an element of hybrid photovoltaic devices [20]. In a typical synthesis, 10.3 mmol of zinc (II) acetate dehydrate, 1.95 mmol of manganese (II) acetate tetrahydrate, and 7.40 mmol of cysteamine hydrochloride were dissolved in 125 mL of distilled water (solution I) under stirring for ~15 min. Simultaneously, 7.4 mmol 4-dodecylbenzenesulphonic acid was dissolved in 2 mL of tetrahydrofuran (solution II) and 5.80 mmol of sodium sulfide was dissolved in 50 mL of water (solution III), respectively. Solution I was placed in a 250 mL three-neck flask. Solution II was very slowly injected under stirring into solution I. To precipitate the ZnS:Mn QDs, the aqueous solution of sodium sulfide (solution III) was slowly added dropwise under stirring into the reaction mixture inside the round-bottom flask. Finally, the turbid dispersion was refluxed at 85 °C under stirring for 3.5 h. The precipitated nanoparticles were collected by centrifugation at 10,000 rpm for 15 min. Finally, the white powder of the ZnS:Mn nanoparticles was dried in a vacuum oven at 60 °C for 24 h.

From our measurements and observations, the most important information was that the agglomeration of nanoparticles occurs very often. This problem was resolved through the use of a special material known as polyethylene glycol monooctadecyl ether, Brij 78 P, with its parameters presented in Table 3 [28].

By applying the Brij 78 P material, the optical and chemical-physical parameters, especially the refractive index and melting temperature, for the used higher alkane materials in the propagated wave were maintained, as was the response for the influences on optical propagation of light inside of the created structure.

Table 4 presents the cumulative refractive indices of the mixtures of alkanes with Brij 78 P (0.5 wt%) and nanoparticles (1 wt%).

### 2.2. Technology

In this section, the technology of threshold sensor manufacturing based on a biconical optical fiber taper covered in higher alkanes with nanoparticles is presented. For the manufacturing of the biconical optical fiber tapers, the author’s arrangements named FOTET II (Fiber Optic Taper Element Technology) were used [34], as presented in Figure 4.

A detailed description of the manufacturing process are included in the previous articles, hence, it will not be presented in this paper [11,34]. Prepared tapers used for presented threshold sensors are characterized by low losses below α = 0.2 dB in a whole investigated range, elongation L = 20.20 ± 0.05 mm and diameter of the taper waist φ = 14.50 ± 0.50 μm.

**Figure 5:**
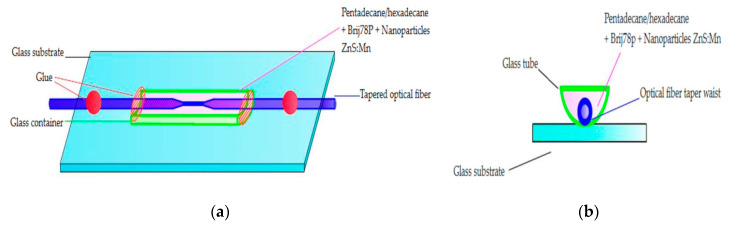

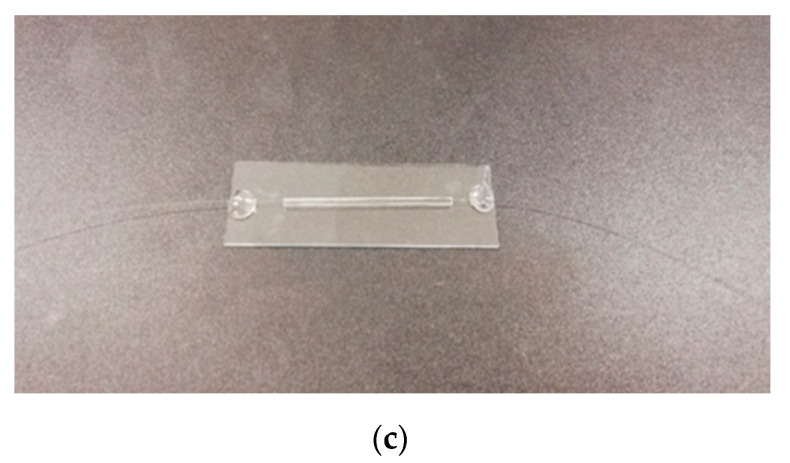
(**a**,**b**) Schematic of arrangements for protection taper with possibilities of alkanes and nanoparticle cladding application, and (**c**) cross-section of a sample.

In the next step, due to the mechanical properties of the prepared taper, the protection tube was used with a schematic and cross-section presented in Figure 5a,b, respectively, and a sample image in Figure 5c.

Measurements were performed for 1550 nm. The choice of this value was dictated by a used optical fiber with the cut off for 1260 nm—a single mode works in a third telecommunication range. Additionally, for this wavelength, all the available measurement equipment possesses the best quality and sensitivity. Furthermore, all provided works are focused on implementation for industrial use. Measurements were provided in stages. The first one was used for spectral analysis and therefore was equipped with a wide range diode type SLED from Excalos (Schlieren, Switzerland) with a range of 1550 nm ± 100 nm and an OSA-AQ6375 optical spectrum analyzer (Yokogawa, Tokyo, Japan). The second measurement system was designed to measure the hysteresis of the temperature change time response. An S3FC1550 single-mode laser source for 1550 nm from Thorlabs (Newton, NJ, USA) was used and a PM100D detector from Thorlabs with a S140C photodiode power sensor was applied. All temperature changes and their stabilization were provided by a climatic chamber VCL7010 from Votche Ltd., (Balingen, Germany).

## 3. Results and Discussion

**Figure 6:**
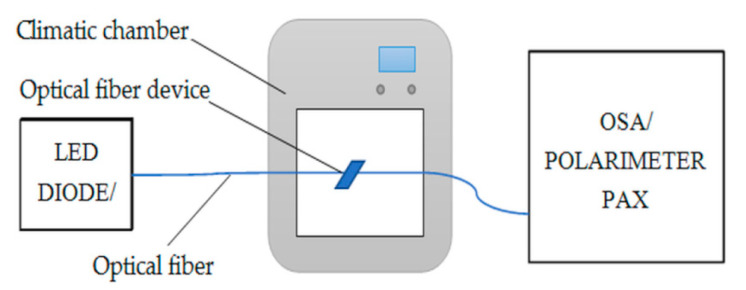
Setup for measurement of optical fiber devices.

The measurements were divided into a few steps, which clearly show the influence of materials that create the taper cladding with different admixtures. The first step provides the manufacturing of an optical fiber adiabatic taper with low insertion losses below 0.2 dB in the whole range. A taper secured in a glass tube was measured in the climatic chamber for a SLED light source, as well as the polarization parameters (Figure 6).

**Figure 7:**
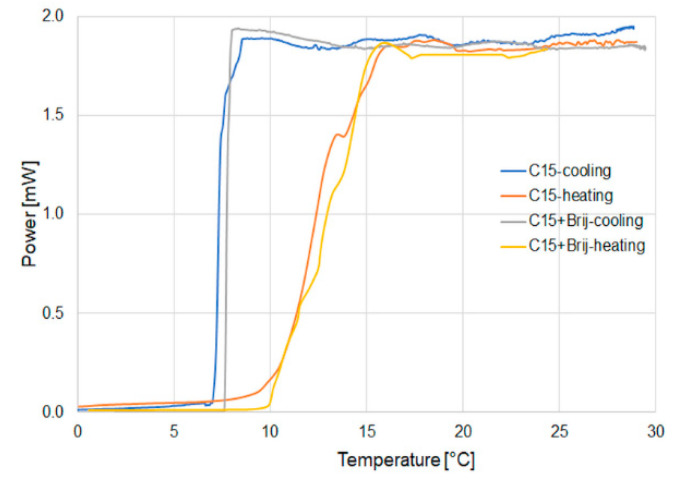
Hysteresis of power change for a pure material and with the Brij admixture for the example of C15 materials.

C15 and C16 were investigated in the temperature range corresponding to the two-phase state—solid and liquid. For the solid phase in both cases, optical transmission in the fiber sensor was very low. This is connected with the value of the refractive index in the solid state and, as well as with the properties of materials, they are not transparent, which causes strong scattering of light by kinds of microstructures within the solid state of all power from the optical fiber taper. At the transition temperature, sharp increases in power are observed and the power is maintained in the liquid state. The additional material Brij 78 P was applied to provide non-agglomeration of the nanoparticles. Figure 7 shows the hysteresis of power change for both pure materials and for those with the admixture of Brij 78 P.

As can be observed, there exists a hysteresis in the cooling and heating process. In all cases, it can be observed that the transmission of light in the heating process is slower than in the cooling one. Additionally, the difference in temperature in which full power is being observed to the temperature it starts to see light transmission is changed from 3 to 8 °C. For the cooling process, the change of power exhibits a step change from maximum to zero. The hysteresis relates to the volume of the materials used. The heating process starts from the outer edge and spreads to the optical taper materials. A full switching range can be observed over 5 °C (from 10 to 15 °C), which is connected with the uneven process of melting materials. In the change of power during the cooling process, levels can be described as a step change. The change from the liquid state to the solid state is 1 °C (8–7 °C). This phase transition is much faster than in the heating process.

In all cases, Brij 78 P does not influence significantly the heating process, as well as the cooling one, so it can be accepted that there is no change of the base material properties. The next part of the investigation relates to the admixture of nanoparticles from ZnS:Mn to both materials.

**Figure 8:**
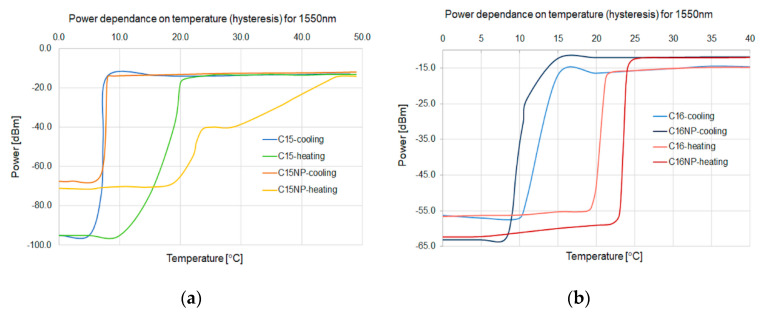
Temperature hysteresis of C15 (**a**) and C16 (**b**) for pure materials and with nanoparticle changes for a 1550 nm light beam.

Figure 8 shows the hysteresis of the power change for a 1550 nm laser (S3FC1550 from Thorlabs) for C15 materials with Brij and with ZnS:Mn is presented.

For both C15 and C16, the hysteresis of a phase change from solid to liquid, and vice versa, can be observed. C15 in the heating process has a much longer time of full change of the physical state. For C16, the time of a physical state complete change is similar for the heating and cooling process. An admixture of nanoparticles for both alkanes changes the temperature of phase transition from solid to liquid one: C15 from 20 to 25 °C and C16 from 22 to 24 °C. This is related to the heating capacity of the used nanoparticles. Furthermore, for the C15 material, a reduction in the phase change from solid to liquid from 10 to 5 °C can be observed. For the C15 alkane with nanoparticles in a heating process, the maximum value very slowly reaches 40 °C.

**Figure 9:**
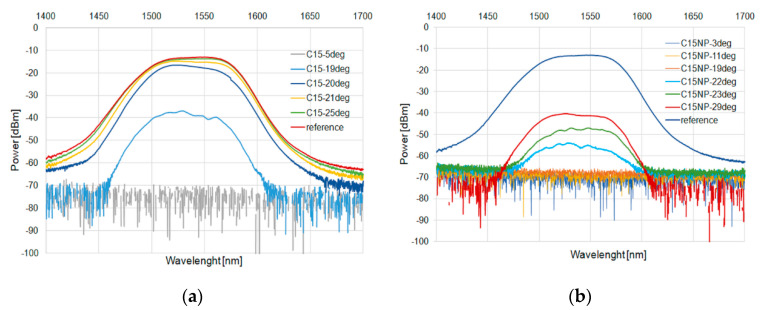
Heating process in C15 (**a**) and with an admixture of NPs (**b**) for SLED 1400–1700 nm.

**Figure 10:**
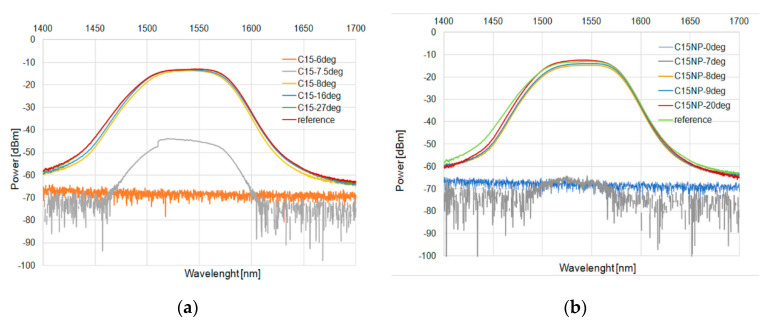
Cooling process in C15 (**a**) and with an admixture of NPs (**b**) for SLED 1400–1700 nm.

**Figure 11:**
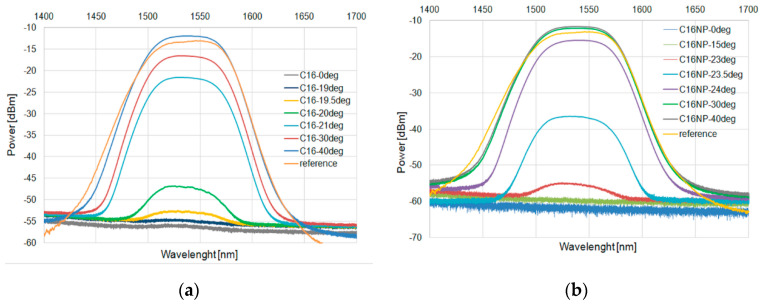
Heating process in C16 (**a**) and with an admixture of NPs (**b**) for SLED 1400–1700 nm.

**Figure 12:**
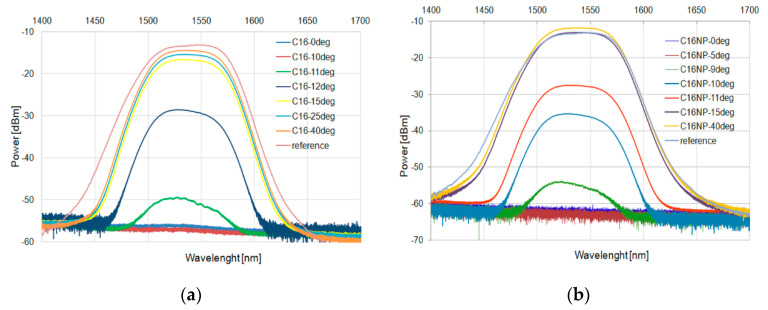
Cooling process in C16 (**a**) and with an admixture of NPs (**b**) for SLED 1400–1700 nm.

These results were confirmed for a wide-range diode. Figure 9 and Figure 10 for C15 and Figure 11 and Figure 12 for C16 present the changes of power in a wide range for the heating and cooling processes, respectively.

In a wide range, the measurement for a single wavelength was confirmed. The presented devices can work for different wavelengths with the same level of temperature detection. Nanoparticles of ZnS:Mn introduce a new kind of nucleating agent, which absorbs some part of the temperature energy (with a higher heat capacity than alkanes) and allows for shifting of the phase state. Moreover, the nanoparticles do not interact with the light propagated inside the taper’s structure but change the properties of the cladding materials.

**Figure 13:**
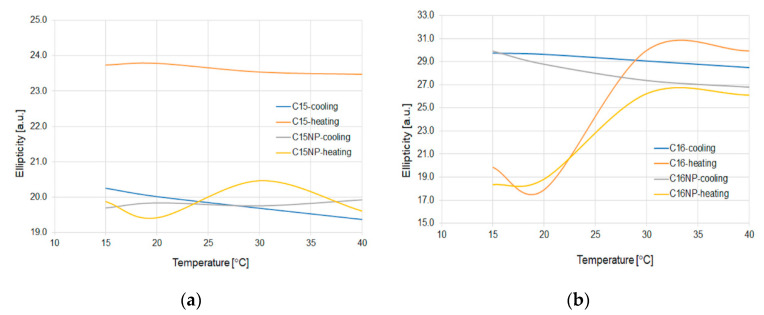
Fluctuation of ellipticity for C15 (**a**) and C16 (**b**) for pure and with NP mixture at 15–40 °C for all microdevices.

For checking the influence of the applied nanoparticles for light parameters, the polarization properties have been checked. As a source, the Santec TLC 210 laser was used for a wavelength of 1550 nm, which corresponds to the telecommunication range and for polarization parameters, the polarimeter PAX from Thorlabs with measurement head for IR wavelength was used. Figure 13 shows the change of ellipticity for the C15 and C16 alkanes depending on the temperature change.

As can be observed for C15, the addition of NPs does not change the polarization properties, especially ellipticity. Nanoparticles cause a situation where there is no change of the heating and cooling value in ellipticity. For C15 with and without the admixture, the change of ellipticity and azimuth is negligible.

For C16 at a lower temperature (below 20 °C), the pure material and the admixture of NP in the heating process do not propagate power that is confirmed by the results—ellipticity and, especially, azimuth are the only measurement on random noise as there is no light. In the cooling process for both types of materials to 15 °C, the state of polarization is maintained. This is strictly connected with the material properties and the value of hysteresis (for cooling process temperature in which power disappears is below 15 °C).

**Figure 14:**
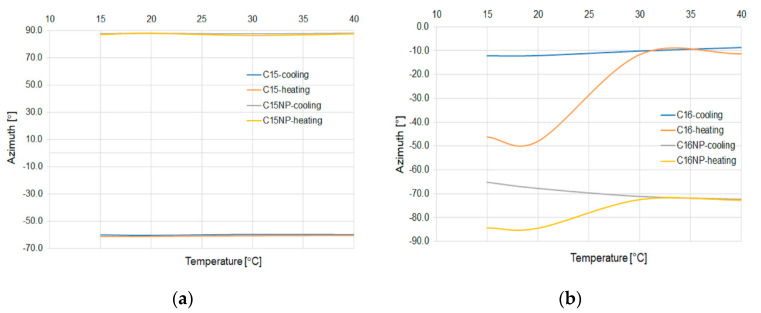
Change of level of azimuth for different temperatures for all kinds of microdevices for pure materials and with NP mixtures C15 (**a**) and C16 (**b**).

Figure 14 shows the change of azimuth for the C15 and C16 alkanes depending on the temperature change.

As can be noticed in a stabilized temperature value of azimuth for both alkanes and their mixtures light power does not change its value. For C15, the value of azimuth does not change during the temperature change. For C16, in the heating process, the value of azimuth increases. In this case, the admixture of nanoparticles reduces this phenomenon.

All observed fluctuation refers only to the climatic chamber properties. For all materials, the value of ellipticity is ±5°.

## 4. Conclusions

In the study, the influence of the admixture of ZnS:Mn nanoparticles to n-pentadecane (C15) and n-hexadecane (C16) has been presented. As can be noticed, the nanoparticles shift the temperature of the change phase state from state to liquid for 3 °C for both alkanes. For the cooling process, it can be observed that the temperature shifts by ~3 °C for C16 to a higher temperature, and for C15, the temperature stays at the same point. These shifts can be a result of the higher heat capacity of the mixture with nanoparticles compare to the pure material. It is noteworthy that it works for a wide spectral range of the propagated light. It is possible to obtain a threshold temperature sensor by admixing NP possess different materials properties. As can be noticed nanoparticles do not introduce a significant change of polarization parameters. For C15, the ellipticity with the NP admixture shows that it changes only in a very small range of less than 1% for the cooling and heating process, for pure material observed the change is over 5%. As can be observed for both alkanes and mixtures with NP, the azimuth stays at the same level independently to temperature and in this case forms a stable polarizer in a wide range. Furthermore, it should be mentioned that in C16 with nanoparticles, we observe standard or pure effects of temperature influence on an SOP parameter of light which is not observed for the second alkane with the nanoparticle, especially in a heating process. The admixture of nanoparticles reduces the change of azimuth in this process to about 10° from 40° for a pure one. For C15 with and without nanoparticles, azimuth stays at the same level. For all measurements, there is no visible fluctuation of polarization parameters during the investigation, which makes the noise ratio of these microdevices negligible.

In all cases, the presented solution can be used as a temperature sensor/detector in difficult to reach places. Additionally, by a special admixture of nanoparticles, it is possible to create sensors that give a signal that the temperature is exceeded above the allowable enabled to react and avoid the damage of equipment or infrastructure. All the proposed solutions are compact, in line, and there is a possibility of using different kinds of lasers.

## Figures and Tables

**Table 1:** Parameters of applied standard telecommunication fiber.

Parameter [Units]	Core	Cladding	Mode Field
Diameter [µm]	8.2	125	10.98
Refractive index [a.u.]	1.4548	1.443	--

**Table 2:** Chemical parameters of n-pentadecane and n-hexadecane [36,37].

Alkane	Formula	Molecular Mass	Melting Point	Boiling Point	Flash Point	Density	Refractive Index	Refractive Index at 20.4 °C
n-Pentadecane	CH_3_(CH_2_)_13_CH_3_	212.42	9–10 °C	269–270 °C	132 °C (269 °F)	0.769	1.4320	1.4317
n-Hexadecane	CH_3_(CH_2_)_14_CH_3_	226.45	18 °C	287 °C	135 °C (275 °F)	0.773	1.4345	1.4344

**Table 3:** Chemical parameters of applied anti-agglomeration material Brij 78 P [29,30].

Formula	Molecular Mass	Melting Point	Solubility
C_58_H_118_O_24_	1199.57	~56–60 °C	At 20 °C in methanol, chloroform and ethanol

**Table 4:** Refractive indices of investigated mixtures at 20.4 °C.

C15	C15 with Brij	C15 with Brij and Nanoparticles	C16	C16 with Brij	C16 with Brij and Nanoparticles
1.4317	1.4319	1.4319	1.4344	1.4346	1.4346
